# Setting health research priorities using the CHNRI method: II. Involving researchers

**DOI:** 10.7189/jogh.06.010302

**Published:** 2016-06

**Authors:** Sachiyo Yoshida, Simon Cousens, Kerri Wazny, Kit Yee Chan

**Affiliations:** 1 Department for Maternal, Newborn, Child and Adolescent Health, World Health Organization, Geneva, Switzerland; 2 Department of Infectious Disease Epidemiology, London School of Hygiene and Tropical Medicine, London, UK; 3 Centre for Global Health Research, the Usher Institute for Population Health Sciences and Informatics, the University of Edinburgh, Edinburgh, Scotland, UK; 4 Nossal Institute for Global Health, University of Melbourne, Melbourne, Australia

**Figure:**
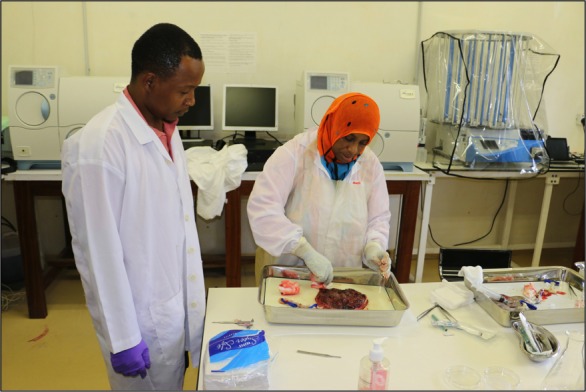
Photo: Researchers in Bangladesh working in their laboratory (Courtesy of Dr Ozren Polašek, personal collection)

Large groups of researchers who agree to offer their research ideas and then score them against pre–defined criteria are at the heart of each CHNRI priority–setting exercise. Although the roles of funders and other stakeholders are also very important, much of the exercise is focused on selecting and engaging a large group of researchers, obtaining their input and analysing it to derive the initial results of the process. In a sense, a CHNRI exercise serves to “visualise” the collective knowledge and opinions of many leading researchers on the status of their own research field. Through a simple “crowdsourcing” process conducted within the relevant research community, the CHNRI approach is able to collate a wide spectrum of research ideas and options, and come to a judgement on their strengths and weaknesses, based on the collective knowledge and opinions of many members of the research community. In doing so, it provides valuable information to funders, stakeholders and researchers themselves, which is obtained at low cost and with little time necessary to conduct the exercise.

Success in involving researchers within each research community, and ensuring their voluntary participation and engagement, is therefore essential to the successful completion of a CHNRI exercise. Over the past few years, we have been involved in assembling groups of researchers to participate in several CHNRI research priority–setting exercises. In this paper, we share our experience of what works well and what works less well and try to answer the most frequently asked questions when it comes to engaging researchers in the CHNRI exercises.

**Figure 1** shows where within the CHNRI process researchers should be involved –which is after the funders have provided their input, and before other stakeholders are approached and asked to contribute.

**Figure 1:**
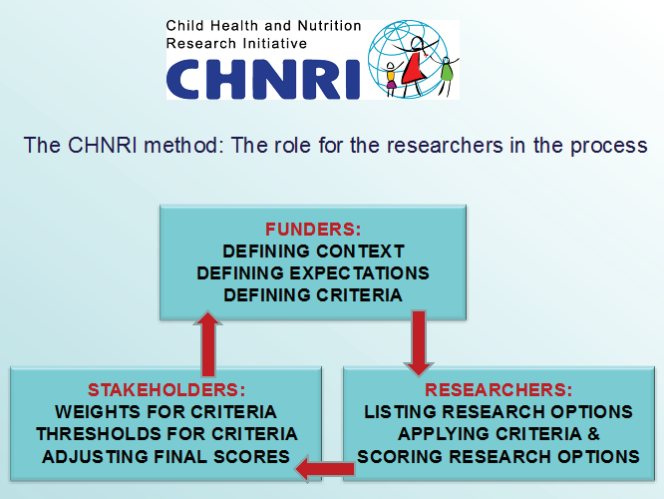
The role of researchers shown within the broader CHNRI process.

## WHY DO RESEARCHERS NEED TO BE INVOLVED IN THE CHNRI EXERCISE?

Following input from funders, as described in a previous paper of this series [1], the managers of the CHNRI process then need to involve a sufficiently large sample of researchers. We discuss the considerations relevant to the optimal size of this sample of researchers in another paper of this series [2]. Researchers have two important roles in the CHNRI process: (i) providing the managers with a broad spectrum of research ideas, which usually span the spectrum of “description”, “delivery”, “development” and “discovery” research; and (ii) providing their own judgement on the likelihood that each submitted research idea will meet a set of pre–defined criteria. These judgements allow the ranking of a large number of submitted research ideas.

At this point, we should explain why CHNRI uses only researchers to provide research ideas, and not other groups of people–eg, funders, programme leaders and managers, other stakeholders, or simply members of the public. This is typically justified on the grounds that researchers are expected to possess far more knowledge and understanding of the state of their research field and the questions that have real potential to generate new knowledge. Importantly, their judgement of each research idea against the priority–setting criteria will also be based on an understanding of the realities of the research process and the success rate in their field. Including participants without this prior knowledge would likely introduce “random noise” into the exercise, resulting in most or all of the ideas receiving similar scores. Thus, restricting participation in these steps to researchers is expected to improve discrimination between the competing research ideas by using the collective knowledge and opinion of a small group of very knowledgeable people.

There is also a practical reason for this: by selecting the most productive, or highly cited researchers over the several preceding years, we are targeting the very group of people who will be most competitive for the research grant calls and likely be awarded the majority of the grants in the immediate future. We should also stress that this is, potentially, a “double edged sword”, because researchers may not be entirely objective in their scoring and may tend to score highly their own preferred areas. This is why the chosen group always needs to be large enough, to prevent anyone's individual input to have a substantial effect on the overall scores. Therefore, the leading researchers are given power through this method to influence the priorities and shape the topics for the future grants, ie, influence the subjects of the calls that are advertised by the funders, rather than simply responding to them. This could also be helpful to the funders, who do not have an easy access to a collective opinion of their research field.

It is worth bearing in mind that an important characteristic of the CHNRI method is its flexibility. Suggestions provided in the guidelines are not prescriptive, and each exercise can be tailored to meet the specific needs of the exercise. For example, some exercises may be mainly focused on implementation (“delivery”) or fundamental (“discovery”) research, particularly if the exercise is related to a specific intervention or geographic context. There have been several examples of such exercises, eg, the implementation of zinc interventions [3], implementation research for maternal and newborn health [4], emerging (discovery–based) interventions for childhood pneumonia and diarrhoea [5,6] and others. In such cases, there is scope for involving further groups of people whose knowledge and experience can provide informative input, particularly if this input is limited to the certain priority–setting criteria where the researchers would be unlikely to possess any first–hand knowledge. For example, hundreds of programme managers contributed to the scoring of questions on the newborn research agenda in relation to its deliverability, affordability and sustainability [7]. Our analyses of previous exercises have shown that the researchers tend to be less optimistic than programme managers on the criterion of answerability, while they tend to be more optimistic on the criterion of deliverability, affordability, sustainability and maximum potential for burden of disease reduction; similarly, programme managers tend to prioritise implementation research questions, whereas researchers prioritised technology–driven research [2,8]. Clearly, a good understanding of the complexities and challenges involved tends to make the experts–whoever they are–more cautious about the prospects of the suggested research ideas.

## HOW TO INVOLVE RESEARCHERS IN THE CHNRI EXERCISE?

In planning the involvement of the group of researchers, the minimum target sample size needs to be decided early in the process. The optimal number will be derived based on the analyses conducted by Yoshida et al. [2], as mentioned previously. Yoshida's analyses suggest that the ranking of proposed research ideas, relative to each other, stabilises at surprisingly small sample sizes–ie, once that 30–50 people with private knowledge on the topic are involved, it is unlikely that the ranking of proposed research ideas will change markedly with the addition of further researchers and their opinions. Given this finding, targeting sample sizes of 50 or greater should result in a replicable CHNRI priority–setting exercise [2].

However, in planning the number of scorers needed, an important issue needs to be considered, which can reduce not only the actually achieved sample size quite substantially, but also introduce potential bias that can invalidate the entire exercise. This is the issue of *(self–)selection bias*. The nature of CHNRI process means that researchers are usually invited (using e–mail or other means) by the management team to take part in the exercise. Their participation is needed in two consecutive steps of the process: (i) providing research ideas that they think would stand a good chance against all other ideas, given the pre–defined priority–setting criteria; and (ii) scoring a long list of research ideas against the pre–defined criteria. While the first step, providing research ideas, is not very time–consuming for researchers, the second step is a lot more time consuming and it may require several hours of input.

In an analysis of the first 50 CHNRI exercises, in which more than 5000 scorers were approached, Rudan et al. reported that the initial response rate (ie, submitting research ideas) was about 60%, with each expert submitting an average of about 3 research ideas. However, when all the initially invited experts were approached again to score the “consolidated” list of research ideas, the response rate dropped to only about 35%. Thus 40% of potential scorers are lost at the first stage, and further 25% of the total number are lost at the second stage (Rudan I, personal communication). The reason for re–contacting everyone who was initially invited to participate, even if they didn't offer any research ideas, is that there may be experts who are not keen giving away their ideas, but would be prepared to score ideas generated by others. This may help to preserve the initial sample that was contacted to the maximum extent possible.

Non–response has two important implications for an exercise. First, it reduces the actual sample size. This can be accounted for–eg, if the desired sample is 100 scorers, then about 300 probably need to be invited to participate in the exercise. Second, and more worrying, is the potential for bias in the results if responders and non–responders differ in their opinions. Results based on inputs from only about one third of the initial pool of researchers contacted may suffer from self–selection bias. For example, if individuals are more likely to respond to an invitation from the management group if they know the members of that group well, they may also be more likely to share similar views with the management group members. Others, who may disagree with those views and may, in fact, be in a majority in that particular research community, would not have their opinions recorded, or would be underrepresented. The high proportion of non–responders in many CHNRI exercise is therefore an important issue and we plan to conduct further work to explore non–response in previous exercises by comparing the characteristics of responders vs non–responders. The important thing to realise in relation to this self–selection bias is that it cannot be attenuated or controlled by further increasing sample size with new invitees because, no matter how large the sample size, they may still be based on the opinions of an unrepresentative subset of research community. In summary, increasing the achieved sample size can be done by inviting more people to participate, or by improving the response rate. The former approach will not attenuate possible self–selection bias, while the latter would tend to reduce the scope for bias and should be preferred. Several reminders are, therefore, usually sent to all invited participants to maximise the response rate.

## SELECTING AND APPROACHING THE RESEARCHERS

The approach to identifying whom to invite to participate in the exercise can be very flexible, but must be credible to both the reviewers of the resulting publication, and also to any researchers who are left out of the exercise (ie, don't get an invitation). We present three examples of previous CHNRI exercises to examine how different strategies may work in different specific situations.

## EXAMPLE OF THE CHNRI EXERCISE ON RESEARCH PRIORITIES FOR CHILDHOOD PNEUMONIA MORTALITY REDUCTION

This exercise [9], published in 2011, involved a small community of researchers working on childhood pneumonia in the low– and middle–income (LMIC) settings. A search for publications on childhood pneumonia in low–resource settings over the previous 5 years listed by the Web of Science identified only a few hundred publications in total. Ranking the authors of these publications ranked by the number of those papers that they had co–authored, revealed that the 100 most productive names were associated with a large majority of papers, and that those authors who were not among the most productive 100 had each contributed 3 papers or fewer over the previous 5 years. The decision was therefore taken to invite the most productive 200 researchers on the basis that this would cover almost the entire research community on this topic, regardless of the nature or importance of their discoveries.

It was agreed that an official approach through the World Health Organization (WHO), that agreed to serve as the hosting hub for the management group, would be most likely to persuade invited researchers to participate in the exercise. Moreover, mentioning that they were selected based on their placement among the 200 most productive researchers in this field would help to make them feel appreciated and that their work is valued. Nevertheless, even with these measures taken, the final response rate in terms of scoring in this small research community was 45/200 (22.5%).

Initially, the researchers were contacted through individual e–mails sent from the WHO, which explained the aim of the exercise, acknowledged the contribution of each researcher to the field, and explained the type of the research idea that was sought–ie, neither too broad, nor too specific (this was further explained in the guidelines for implementation of the CHNRI method) [10]. They were also asked to consider all types of health research, ie, “description”, “delivery”, “development” and “discovery” and they were given an example of a “valid” research idea from each of those four types of research. They were initially given up to one month to submit as many research ideas as they wished, and two further reminders were sent at two weekly intervals following the initial deadline before the total number of submitted ideas reached 500. At that point, reminders were stopped and the management group studied the potential bias introduced because some researchers submitted many more ideas than others. At that point, a “consolidation” of the list of research ideas was conducted to ensure that the retained questions are evenly distributed across different research instruments and main research avenues and cover them all reasonably well. In this phase, all duplicate ideas were removed, while similar ideas were compressed into a single research question. This resulted in the reduction of the number of research ideas considered for scoring from 500 to 158, thus also making the scoring process more manageable.

Depending on the number of research ideas and the anticipated time required for scoring, one option is to offer the scorers the option of only scoring the criteria that they feel most comfortable with scoring–another flexibility in the CHNRI method. It is important that each scorer scores all research ideas on the same criterion, rather than scoring some but not all ideas for all criteria. This ensures that each research idea is scored by the same set of scorers, avoiding any personal preferences towards some ideas and keeping the process transparent and fair.

Given that scoring is time consuming, it was considered reasonable to allow the scorers about a month to reply, with two further reminders sent at monthly intervals after the deadline. After 3 months, the scoring process should typically be considered completed, the drop–out rate recorded, and the analyses can begin. The process of analysis of the scores is described in great detail in another paper [10].

## EXAMPLE FROM THE CHNRI EXERCISE ON RESEARCH PRIORITIES FOR NEWBORN HEALTH

This study has been published in its extended form in this theme issue [7]. Although the field of newborn health in low–income settings is very recent and the research community is still quite small, and although the process of involving researchers followed many steps that were in common to the exercise on pneumonia 5 years earlier, several important innovations were introduced.

Similarly to the pneumonia exercise, the management group selected the 200 most productive researchers, based on the number of co–authored publications in peer–reviewed journals in the previous 5 years. However, the composition of those 200 researchers was more targeted in this case: in addition to inviting the 100 most productive researchers on newborn health globally, the 50 most productive researchers affiliated to institutions in low and middle–income countries (LMIC) were also invited. The final 50 invitations were reserved for the most productive researchers in the area of stillbirth research globally. The purpose of this approach to sampling was to avoid under–representation of researchers from LMIC and the small number of researchers who worked on the increasingly important issue of stillbirths. This was a carefully thought–through approach and is another example of the flexibility allowed in the CHNRI process. It is important to “design” the sampling process in a way that captures researchers who could be most informative for the specific exercise, which is likely to be more important for exercises that are very broad in scope and less important for those which are very narrow.

Another innovation in this newborn health exercise was the inclusion of programme managers, identified through the Healthy Newborn Network database. This was a suggestion made by several members of the management board in light of broad agreement that “description” research was no longer a priority and that the new focus should be on implementation. Therefore, the group recognised the need to include experts with first–hand understanding of the challenges with delivery, cost and sustainability of newborn health and stillbirth prevention programmes in LMIC settings. This resulted in about 600 potential scorers being invited to participate in the exercise, of which the majority (400) were program managers familiar with the challenges in low–resource settings. Eventually, 132 persons participated in the generation of ideas and 91 in scoring, bringing the final response rate to about 15%.

Another innovation in this exercise was the use of “Survey Monkey”, which allowed the management group to keep track of the age, gender, geographic area, background and affiliation of each participating researcher/programme manager in real time. This innovation was seen as very useful, because it allowed more intense reminders that were being sent to specific groups of invitees who were falling behind and becoming under–represented.

To improve the response rate, the management team sent four and five reminders to the invitees for both submitting the ideas and the scores. The team met in Geneva for a week to consolidate the initial list of research ideas they had received from about 400 down to about 200 that were eventually scored. In summary, this exercise stands out in three ways: (i) the targeted sampling of researchers; (ii) the inclusion of programme managers as the majority of invited scorers, to better reflect the community with useful knowledge on the criteria, which is not necessarily reflected in academic articles; and (iii) the tracking of score responses in real time using survey monkey [7].

## EXAMPLE FROM THE CHNRI EXERCISE ON RESEARCH PRIORITIES FOR DEMENTIA

The examples on childhood pneumonia and newborn health are both relevant to research fields with relatively small research communities. In both exercises, the CHNRI method was used primarily as a way to galvanise the community and define the strategy for the development of the field. The small number of productive researchers in both fields meant that nearly everyone who had contributed to the research field over the previous 5 years was invited to participate in the exercise. However, how should we select researchers when the research field is very large and has tens of thousands of actively participating researchers? One such recent example is the CHNRI exercise on dementia and Alzheimer disease, a field in which tens of thousands of researchers are active. This exercise represents a good example of the strategies that can be used to solicit input from researchers in such circumstances.

The management group numbered 15–20 members at various stages of the process and included representatives of the World Health Organization, several international societies and funders interested in this topic (eg, Alzheimer Disease International, USA–based Alzheimer Association, UK's National Institute for Health Research, Canadian Institute for Health Research and USA–based National Institute of Aging), together with leading researchers and opinion–leaders in the field who were based in academic institutions (Rudan I, personal communication). This diverse group needed to devise a plan for recruiting a large number of researchers to provide research ideas and scores for the vast multi–disciplinary field of dementia and Alzheimer disease research. They held several meetings and teleconferences during which they discussed the best strategy to address this difficult task.

Their discussions soon focused on finding the proper justification for inviting some researchers, while leaving many thousands of others outside of the exercise. The group started to look for an appropriate response to a likely *post–hoc* question “*Why wasn't I invited to participate, and other colleagues were?”* that would eventually be acceptable to all those who might ask this question. The group eventually agreed that a justification that was likely to be accepted by researchers in this area should have the following format: “*You were not invited because: (i) you were not among the most productive 500 researchers (in terms of the number of publications) in this field in the past 5 years; (ii) you were neither the lead, nor the senior author on any of the 50 most cited papers in each of the past 5 years; and (iii) you don't belong to any of the groups of researchers specifically targeted for inclusion (even if they do not fall into the first two categories); this mainly relates to the few researchers from low– and middle–income countries (LMICs)”*.

Given that the line of whom to invite needs to be drawn somewhere, the CHNRI management group agreed that the justification provided above would have a good chance for being accepted by the entire research community. Indeed, if a researcher isn't among the 500 most productive in the field in the previous 5 years, they cannot easily take an issue over those 500 more productive being invited. Moreover, if a researcher hasn't led the research on a paper that was later ranked among the 50 most cited papers on the topic in each of the 5 previous years, then they cannot easily take an issue over the invitation of those 500 further authors who were in this position (5 years × 50 papers × (1 lead +1 corresponding author) = 500 authors). This rule implied that up to 1000 researchers would be invited to participate–some based mainly on their productivity in this field, and others mainly on high impact of their work, with some overlap expected between the two groups. Finally, given that the exercise was global in terms of geographical scope, and that the vast majority of the most productive and/or cited authors were based in wealthy countries, the group concluded that every effort should be invested to identify the third group to invite–composed of an unrestricted, but likely quite small number of prominent published researchers based in low– and middle–income countries, which would be sought for through a separate effort.

The productive authors for the first group were identified through a search of Web of Sciences' “Core Collection”, which ranked all researchers in the world in the field of dementia or Alzheimer disease by the number of publications, limited to the output in the preceding 5 years (2009–2013). This allowed the CHNRI management group to identify 500 most productive researchers. The group also needed to check and merge results for the same author who published with different initials (ie, interchangeably using only one or both initials in their papers). The contact details were then successfully obtained from their publications for a sizeable subset, although not for all. This potentially introduced a bias related to dropping those who couldn't be contacted from further stages of the process.

The group then used Web of Science’s “Core Collection” to rank the papers published in each of the years 2009–2013 by the number of citations that each paper received by the end of 2014. For the 50 most cited papers in each year, the group identified the lead and the corresponding author (ie, the first and last listed). After removing duplicate entries–because some authors would be found on several such papers, and then also on the previous list of the most productive authors–the identified authors would be invited to participate in the exercise wherever their contact details could be found. All duplicates were removed, but the “new free places” would not be filled with further scientists, because the justifications for inclusions were pre–set and it would not be easily decided whether to keep filling the places based on productivity, citations, or some other criterion. This meant that the final number of invited researchers would decrease from 1000 to a smaller number. Due to the overlap, the described process yielded 672 researchers to be contacted.

In addition, Chinese databases were systematically searched. The papers published in those databases didn't have many citations (as checked through Google Scholar), so the ranking of papers by citations received could not have been used as a selection criterion in a truly meaningful way. The group therefore invited the most productive 50 authors from the Chinese literature over the preceding 5–year period (2009–2013). To identify the few researchers from other low– and middle–income countries, the Alzheimer Association, Alzheimer Disease International (ADI, which is the global umbrella organization of all national Alzheimer associations) and 10/66 dementia research group (broad network of researchers from low and middle income countries) were actively involved in identifying and contacting the experts in LMIC. In the end, about 800 researchers were identified for contact, and the contact details were successfully obtained for 69% of them, each of whom was asked to submit 3–5 research ideas. Then, a total of 201 experts responded and submitted 863 research ideas. Those ideas pertained to prevention, diagnosis, treatment or care for dementia and represented “basic”, “clinical–translational” or “implementation” research, as categorized by the management group. The management group then decided that this number was too large to score, so they convened a meeting to review all received research ideas. They consolidated the list to 59 representative “research avenues/themes”, which were broader than specific research ideas/questions. These broader avenues/themes were then scored using a slightly modified set of the 5 standard CHNRI criteria. Thus, this exercise developed not only an approach to the sampling of experts when a very large number of experts exists in the world, but also developed an approach to deal with an unmanageable number of specific research ideas/questions received from such a large expert group. It is possible that, in the final version of the published paper (which is now still under review), some minor practical modifications from this protocol will be observed (Rudan I, personal communication).

## ETHICAL AND OTHER CONSIDERATIONS

Given that the CHNRI method essentially relies on input from human subjects (who are researchers in this case), we consider here the ethical aspects of conducting CHNRI exercises. The CHNRI exercises are a form of research that uses various measures of collective opinion as an output–eg, the level of collective support for a particular research idea, the extent of agreement within the collective, the variance in all expressed opinions, the average level of support across several criteria, and possibly others. Nevertheless, the input is based on individual opinions received from individual participants.

The method itself, as initially proposed [10], underwent ethical scrutiny at the institution where it was conceived–at the Croatian Centre for Global Health at the Faculty of Medicine of the University of Split, Croatia. The following recommendations were given:

(i) It is important to let all participants know, at the stage of inviting them to participate in the CHNRI exercise, that by responding to the invitation through submitting their ideas, and then their numerical scores, they acknowledge their voluntary participation in the exercise; this will deal with the ethical concern over whether their participation is voluntary, and they would not need to sign a special informed consent;

(ii) Although the input received from the participants is encoded as a sequence of numbers (the scores), if it is presented in the supplementary material of the resulting papers under the scorers' personal names or surnames, and aligned against the research ideas that were scored, this can still be used to reconstruct their personal opinions on a wide range of research topics; this may make the participants (ie, scorers) uncomfortable. Therefore, unless specific approval is obtained at the individual or a group level to disclose all individual scores in the interest of transparency of the CHNRI exercise (which is a motivation that can be seen as being in conflict with ethics concerns in this case), we recommend that all scores disclosed in the public domain through publications should be anonymized. If the scores received from the scorers are anonymized in a proper way, and only the opinion of the entire collective is studied and interpreted, there should not be any ethical concerns related to the CHNRI exercise.

(iii) We see another theoretical ethical concern that should potentially be carefully managed; namely, if all participants and their scores are disclosed in the public domain, and the participants haven't been anonymised at their own request (ie, in the interest of transparency and legitimacy of the CHNRI exercise), then the participants should still be warned that further statistical analyses could potentially be performed on the data set that involves their names. Those analyses could focus on participants themselves as subjects, and “ranking” and comparisons among the participants, rather than research ideas. Therefore, everyone's input could be statistically compared to that of one or more other participants. Although this is never the intention or a focus of the CHNRI exercise, it is a theoretical possibility and it could identify some scorers as “outliers” in terms of scoring with respect to their colleagues, which may cause them an unforeseen concern.

If these theoretical concerns are appropriately addressed and managed, which can most easily be achieved through informing the participants of the scope of the exercise, explaining that by self–selecting themselves for the exercise they are acknowledging their voluntary participation, and anonymising their scores once they are received, the CHNRI method should be considered free from ethics concerns.

The managers of CHNRI exercises often ask whether the results of the exercise should be returned to all participants. We endorse this practice, because we can see no reason why this should not happen. It is in everyone's interest to inform them of the collective optimism/pessimism towards various research ideas within each research community, especially when the participants have freely offered their ideas and time for scoring.

This brings us to another frequent question, which is how to thank the participants for their contributions in terms of suggesting research ideas and dedicating their time to scoring? In the vast majority of the previously conducted CHNRI exercises, this was done through involving the participants in the resulting publication. This involvement could either take the form of equal co–authorship, or listing under the group co–authorship, or simply acknowledging their contribution in the acknowledgement at the end of the paper. The decision as to which of these three options to employ typically depends on the number of participants, the realistic prospects in involving them in other stages of writing of a resulting CHNRI publication (beyond purely providing the scores), and the preferences, restrictions, or authorship criteria of the journals to which the papers have been submitted. It is also possible to motivate the participants to participate in the CHNRI exercise by organising a meeting in a convenient location and supporting participants’ travel and accommodation expenses, and then conduct the entire exercise over a few days in a location of preference or convenience. In some cases, this has been done to expedite the scoring process when speed is important as exercises can take quite a long time when conducted via e–mail [4–8].

## CONCLUSIONS

To date, we have gained a considerable experience with involving researchers as participants who provide research ideas and scores for the CHNRI exercises. We have tried to summarise some informative examples in this paper, irrespective of whether the chosen examples were necessarily the most successfully conducted CHNRI exercises. Indeed, it is difficult to judge whether the CHNRI exercise has been “successful”, and what criteria should be used to do so. Clearly, a high participation rate should limit the scope for response bias (through self–selection), which is a major concern with CHNRI exercises. Then, a large and broadly inclusive spectrum of research ideas provided by participants and made available for scoring would certainly signal a success in conducting the exercise, although it is difficult to quantify this inclusiveness. Moreover, it would reflect researchers' willingness to share their ideas freely and take part in the process. Large differences in the final research priority scores (RPSs) received by various research ideas indicate that the criteria used are able to discriminate between ideas.. If an exercise results in only small differences in RPSs then any ranking of research ideas based on the scores is unlikely to be very robust, and the exercise will have largely failed to meet its own objectives.

Finally, if the exercise is conducted reasonably quickly (typical time is about 3–6 months) and at low cost (typical direct financial costs are up to US$ 15 000, unless the costs of organizing one or more meetings are envisaged), and all participants accept the results and co–author a resulting publication, then the exercise has served its purpose. This will be even more so whenever there is a vision of a follow–up to the exercise, in which a workshop is organised to arrange research proposal writing, or a special meeting with the funders is agreed to ensure that the priorities have been properly communicated. Dissemination of the results and an appropriate follow–up at national, regional and global levels are important parts of the CHNRI process, to increase the likelihood that the research on identified priorities is conducted in the near future. Evaluating whether CHNRI exercises have had an impact on those who invest in health research and influenced investment decisions is challenging and is will be addressed in future papers on the CHNRI method.
